# Supplementary material for: Dynamic Expression of Interferon Lambda Regulated Genes in Primary Fibroblasts and Immune Organs of the Chicken
Source: Genes (Basel). 2019 Feb 14;10(2):145. doi: 10.3390/genes10020145 (PMC6409627; doi:10.3390/genes10020145)
Supplement: Supplementary file 1 [file genes-10-00145-s001.zip › Supplimentary Files/Supplementary Figures (Full and Final).pdf]

A

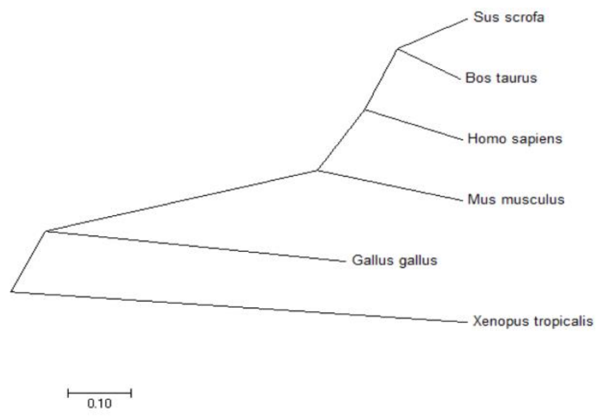
Sus scrofa
Bos taurus
Homo sapiens
Mus musculus
Gallus gallus
Xenopus tropicalis
0.10

B

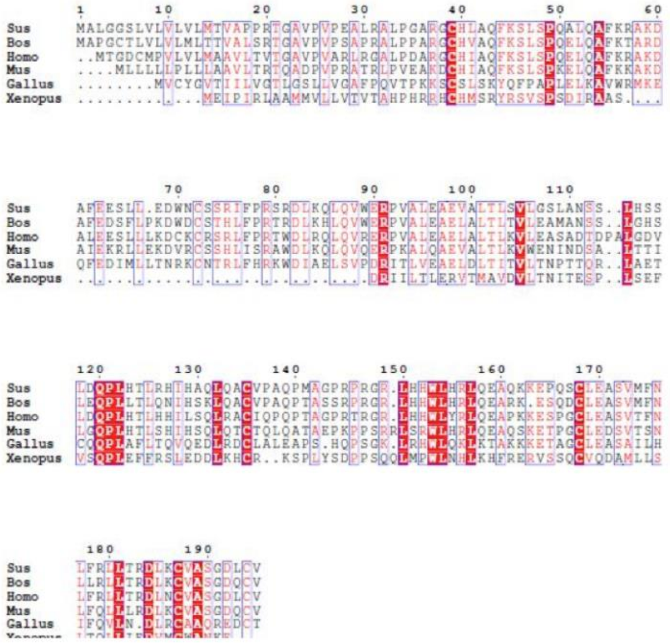
Sus
Bos
Homo
Mus
Gallus
Xenopus

C

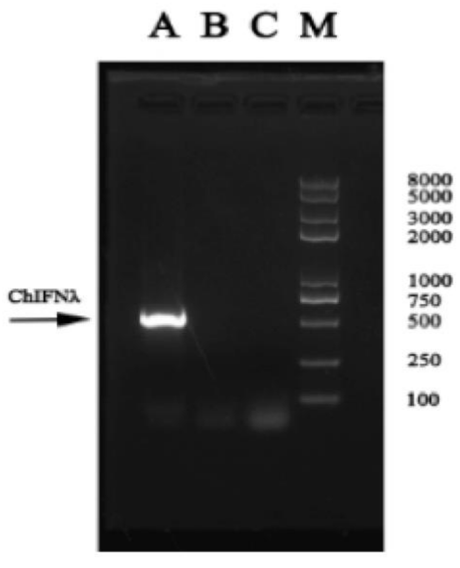
A B C M
ChIFNλ
8000
5000
3000
2000
1000
750
500
250
100

Supplementary Figure 1: A: Phylogenetic analysis representing the relationship of chIFN-λ with vertebrate IFN-λ. The tree was constructed in MEGA 7 by employing neighbor joining method (bootstrap 1000). All gene sequence were acquired from available database (NCBI). Sus scrofa (pig) [NP_001159962.1], Bos taurus (cattle) [NP_001268830.1], Homo sapiens (human) [AAN86127.1], Mus musculus (mouse), Gallus gallus (chicken) [XP_015144667.1], Xenopus tropicalis (frog) [NP_001165236.1]. **B**: Amino acid sequence of multispecies IFN-λ were aligned using ClustaLW algorithm. Red color represents conserved sequences. ESPript 3.0 was employed to analyze the sequences. **C**: PCR validation of chIFN-λ gene expression. Within C section, A) Expression product chIFN-λ cDNA; B) Baculovirus control cDNA; C) Expression product mRNA; M) Marker.

A

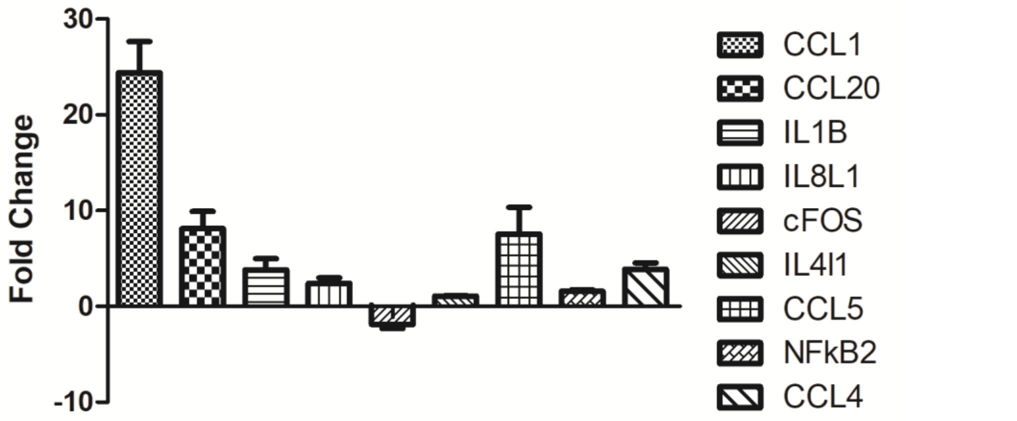

B

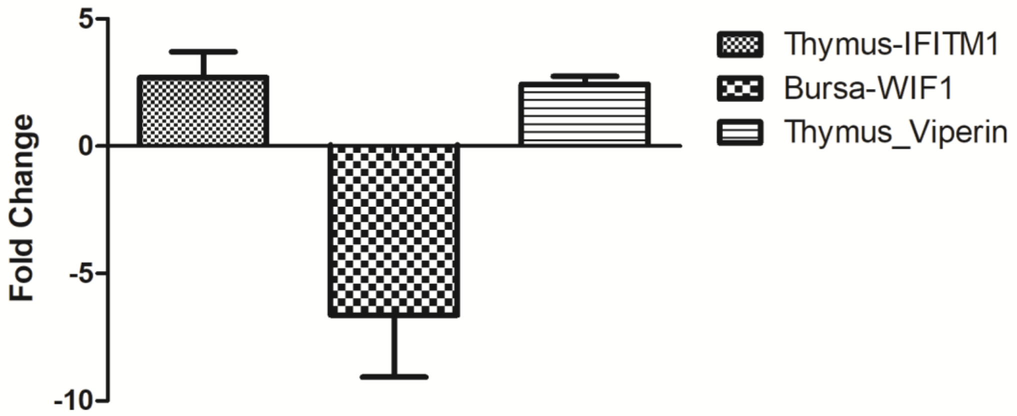

Supplementary Figure 2: A: Gene expression in IFN lambda induced CEF and **B:** Gene expression in chicken tissues.
