# Supplementary material for: Dynamic Expression of Interferon Lambda Regulated Genes in Primary Fibroblasts and Immune Organs of the Chicken
Source: Genes (Basel). 2019 Feb 14;10(2):145. doi: 10.3390/genes10020145 (PMC6409627; doi:10.3390/genes10020145)
Supplement: Supplementary file 1 [file genes-10-00145-s001.zip › Supplimentary Files/Supplementary Figures (Full and Final).docx]

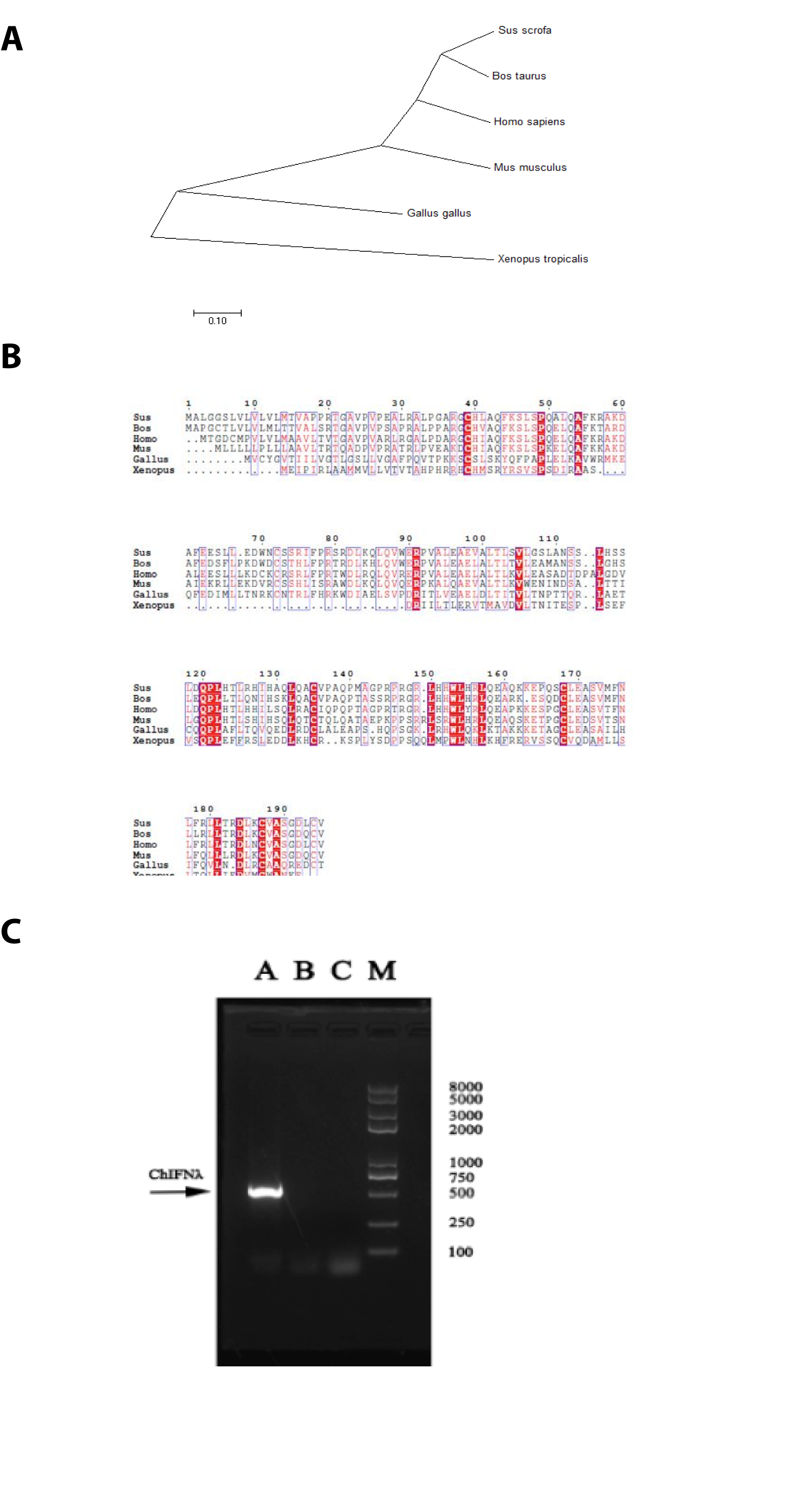


**Supplementary Figure 1: A:** Phylogenetic analysis representing the

relationship of chIFN-λ with vertebrate IFN-λ. The tree was constructed in

MEGA 7 by employing neighbor joining method (bootstrap 1000). All gene sequence were acquired from available database (NCBI). Sus scrofa (pig)

[NP_001159962.1], Bos taurus (cattle) [NP_001268830.1], Homo sapiens (human) [AAN86127.1], Mus musculus (mouse), Gallus gallus (chicken)

[XP_015144667.1], Xenopus tropicalis (frog) [NP_001165236.1]. **B**: Amino acid sequence of multispecies IFN-λ were aligned using ClustaLW algorithm. Red color represents conserved sequences. ESPript 3.0 was employed to analyze the sequences. **C**: PCR validation of chIFN-λ gene expression. Within C section, A) Expression product chIFN-λ cDNA; B) Baculovirus control cDNA; C) Expression product mRNA; M) Marker.

**A**

**
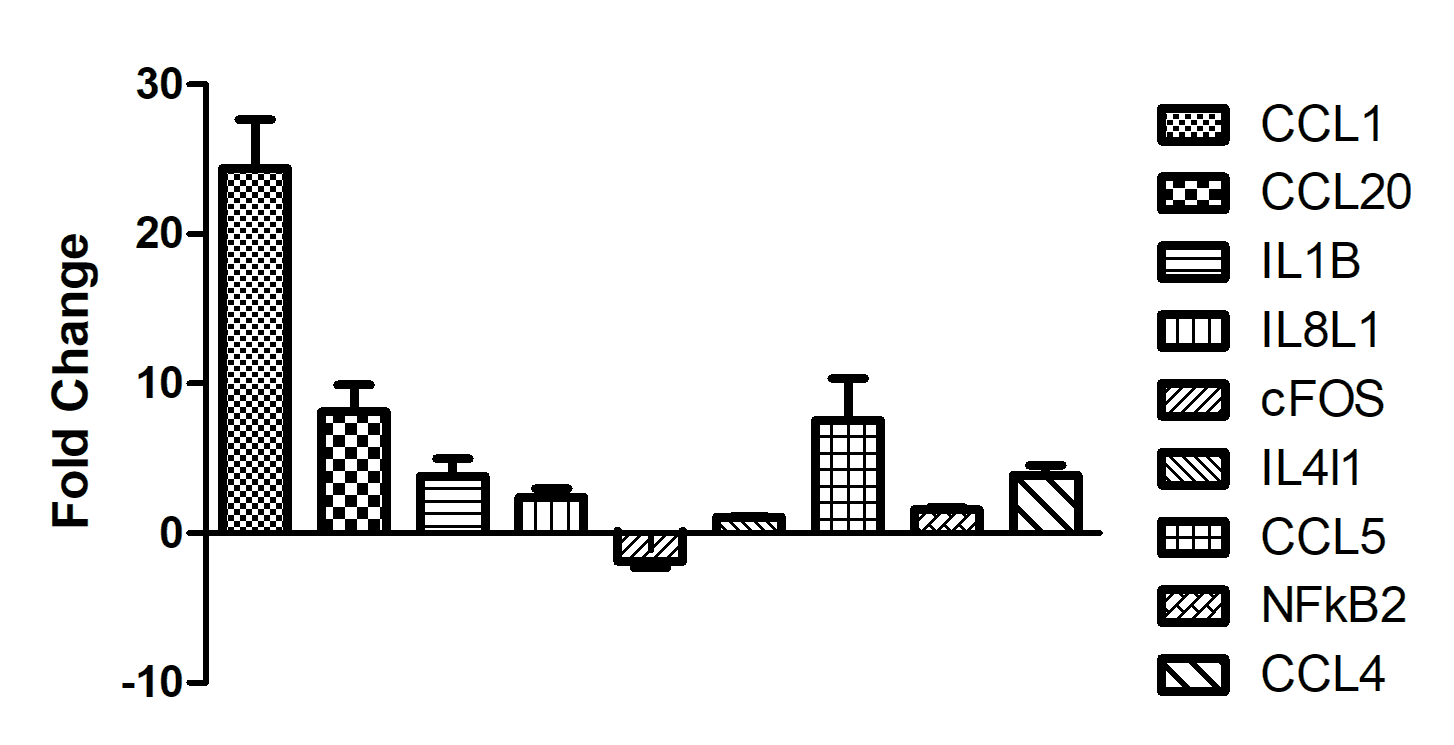
**

**B**


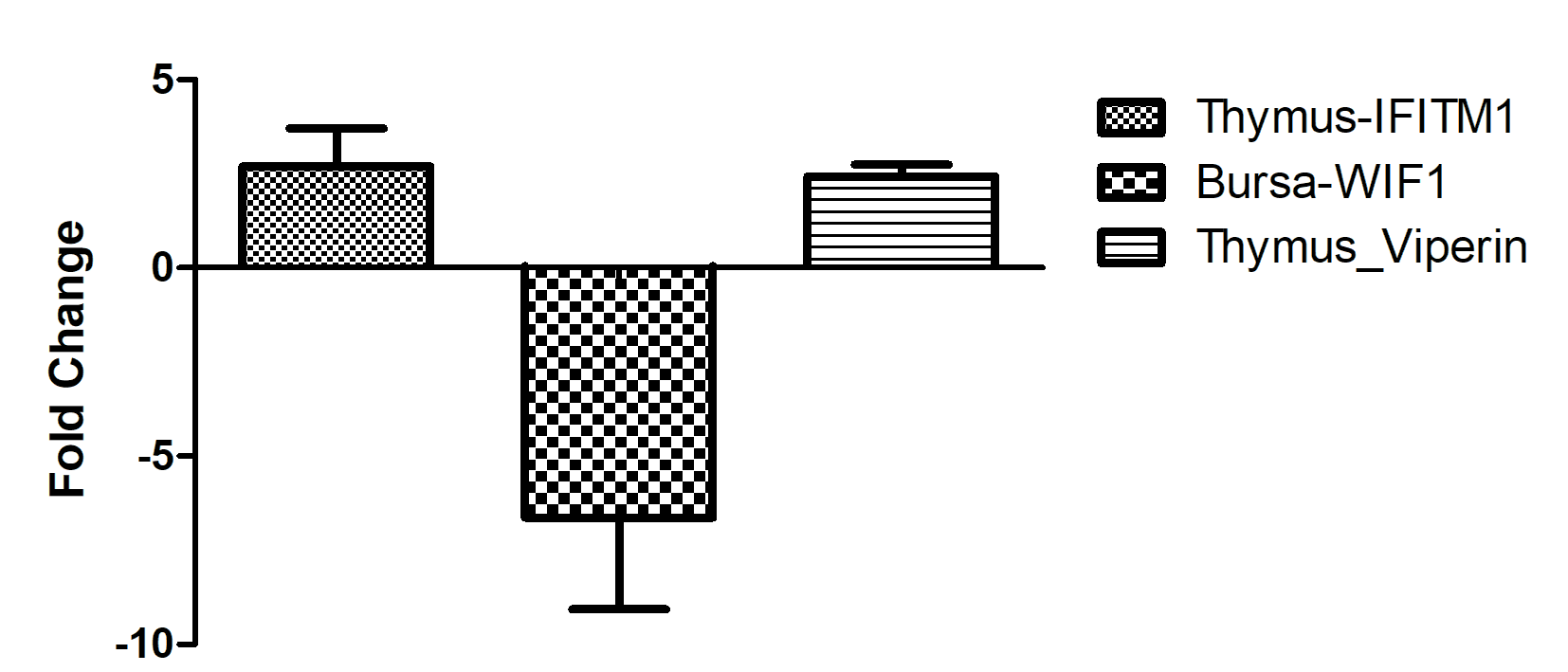


**Supplementary Figure 2: A:** Gene expression in IFN lambda induced CEF and **B:** Gene expression in chicken tissues.
